# Revolutionizing radiology education: exploring innovative teaching methods

**DOI:** 10.1007/s00261-025-05010-x

**Published:** 2025-06-02

**Authors:** Izzet Altun, Ozerk Turan, Omer Awan

**Affiliations:** University of Maryland, Baltimore, Baltimore, USA

**Keywords:** Active learning, Radiology education, Flipped classroom, Teaching methods

## Abstract

The field of radiology education is undergoing a paradigm shift due to technological advancements and the increasing complexity of medical imaging. Traditional didactic teaching methods are progressively being supplemented or replaced by innovative pedagogical approaches that enhance engagement, competency, and clinical preparedness. This review examines the evolution of radiology education, highlighting novel teaching methodologies such as simulation-based training, artificial intelligence assisted learning, virtual and augmented reality, flipped classrooms, and case-based learning. Furthermore, this manuscript discusses the challenges of integrating these methodologies into radiology curricula and explores potential future directions in radiology education.

## Introduction

Radiology is an essential component of modern medicine, playing a critical role in diagnosis, treatment planning, and patient management. The increasing complexity of medical imaging modalities, coupled with advancements in artificial intelligence and digital technologies, necessitates a transformation in radiology education. Traditional models of radiology training, including didactic lectures, textbook learning, and educator lead methodologies, are now being augmented by innovative educational strategies designed to improve clinical decision-making, image interpretation skills, and hands-on proficiency.

The purpose of this review is to evaluate contemporary teaching methodologies that have emerged to enhance radiology education. We will explore the advantages and limitations of various modern teaching approaches, assess their impact on student learning and competency development, and discuss future directions in medical education that can further improve radiology training.

## Bloom’s taxonomy and radiology education

Benjamin Bloom proposed an taxonomy describe cognitive learning levels and categorizing educational goals and objectives which was published in 1956 and revised in 2001 [1]. Bloom’s Taxonomy provides a valuable framework for structuring educational objectives in radiology, progressing from basic knowledge recall to complex creation and innovation.

Bloom’s Taxonomy provides a hierarchical progression of cognitive abilities, spanning from foundational remembering to sophisticated creation, and serves as a fundamental framework in educational pedagogy (Table 1). In the context of radiology, where artificial intelligence is increasingly influential, the cultivation of higher-order thinking skills, as emphasized by Bloom’s Taxonomy, is essential for developing the critical reasoning necessary for accurate image analysis and optimal patient care [1, 2].

**Table 1:**
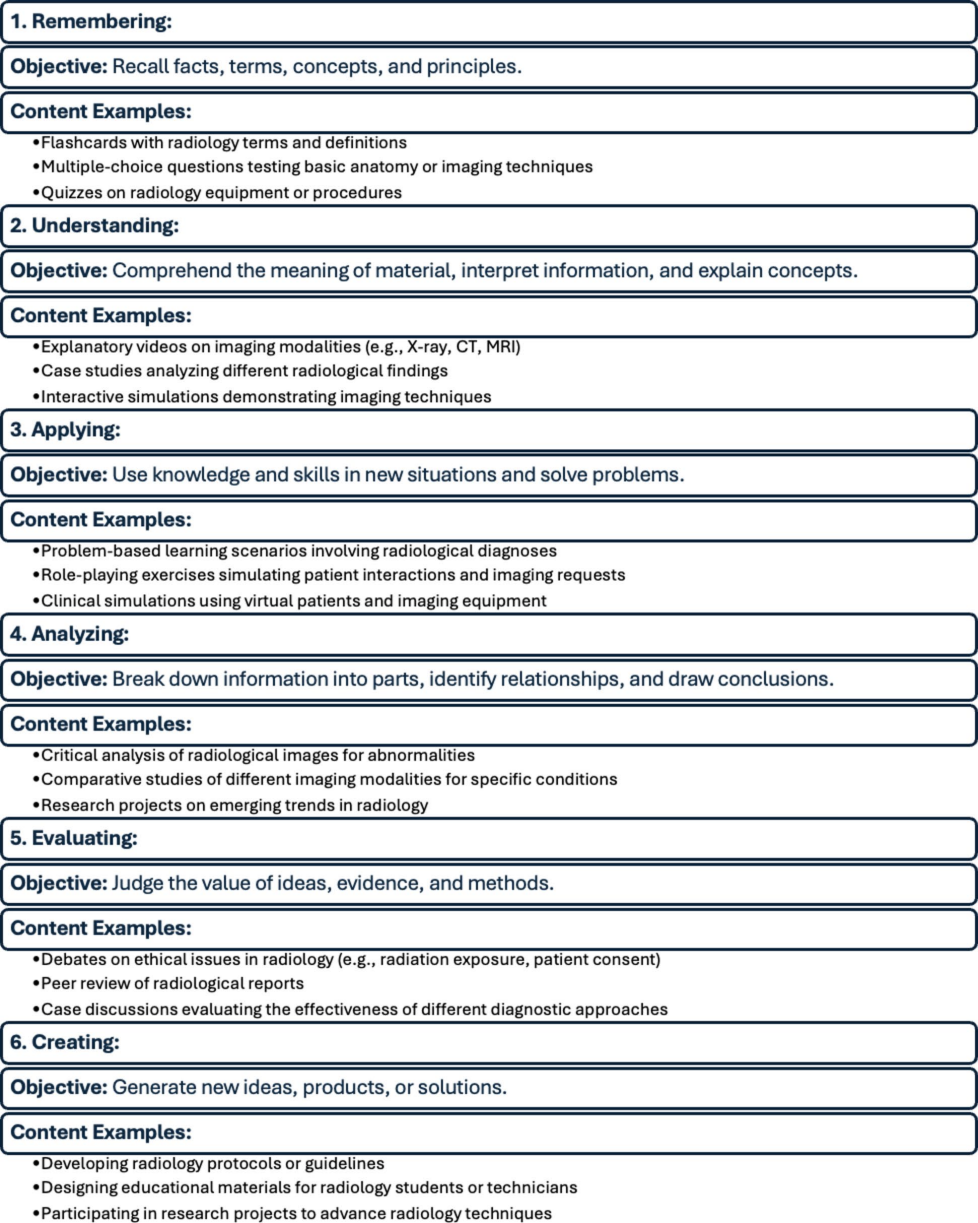
Summary of Bloom’s taxonomy

The first level, Remembering, focuses on the recall of facts, terms, concepts, and principles. This can be supported through the use of flashcards with radiology terms and definitions, multiple-choice questions that test basic anatomy or imaging techniques, and quizzes on imaging techniques or procedures. For second level understanding, learners are expected to comprehend the meaning of material and explain concepts. Effective tools at this level include videos on imaging modalities, case studies that present various findings which were previously reviewed, and interactive simulations.

At the Applying level, residents use their knowledge and skills to solve/interpret the unfamiliar problems. This can involve problem-based learning scenarios centered around interpreting findings in images using their knowledge and ability to recall. This category involves residents daily practice including reading studies trying to figure out diagnosis which they never seen before and make a decision based on their clinical judgement. Also, during case conferences residents use their knowledge and apply it to come up with a diagnosis or make a recommendation based on images available. The fourth level, analyzing, requires learners to break down information, identify relationships, and draw conclusions. This is achieved through critical analysis of radiological images for abnormalities, comparative studies of different imaging modalities for specific conditions, and research on emerging trends in radiology.

In the Evaluating stage, residents are encouraged to criticize. They can engage in debates on issues in radiology such as radiation exposure or patient consent, conduct peer reviews of radiological reports, and participating in case discussions. Finally, the highest level, Creating, involves generating new ideas, products, or solutions. At this stage, residents may develop new radiology protocols or guidelines, design educational materials for students or technicians, and participate in research projects aimed at advancing radiology techniques. This hierarchical approach ensures a comprehensive and progressive development of knowledge and skills essential for effective radiology education.

Radiology education must prioritize the development of critical thinking skills, facilitated by engaging with the higher levels of Bloom’s Taxonomy. Failure to intentionally cultivate these advanced cognitive processes can result in learning deficits, ultimately compromising learners’ capacity to analyze and evaluate essential radiologic data [2]. Although Bloom’s Taxonomy offers a powerful framework for enhancing teaching effectiveness, its practical integration into daily practice is underdeveloped, primarily due to the limited availability of tangible descriptions and examples illustrating its application in real-time clinical settings [3].

## Teaching tools in radiology education

The landscape of radiology education has undergone significant transformation over the decades, following advancements in medical technology and pedagogical understanding. Historically, radiology education has predominantly relied on a set of established methods, each with its inherent strengths and limitations. Didactic lectures structured presentations formed a cornerstone of early radiology education, providing residents with foundational knowledge in anatomy, pathology, and imaging principles. While efficient for conveying large volumes of information to a group, their effectiveness can be limited by passive learning, a lack of real-time interactivity, and challenges in maintaining resident engagement over extended periods. Furthermore, the one-size-fits-all approach may not fit to all type of learning styles and paces of individual residents.

When structuring lectures educators should use Blooms taxonomy to achieve their goals of teaching to residents and review their methods and materials to see which levels of Blooms taxonomy they are addressing. One method to address all the levels in blooms taxonomy using active learning strategies. Active learning strategies are methods to engage learners to actively involve in learning process make them do things and thinking about the things they are doing. There are many active learning methods in radiology education can be used by educators including Flipped Classroom, Case-Based Learning, Peer Teaching, Audience Response Systems (ARS) and Gamification.

### 1—Flipped classroom

**Fig. 1:**
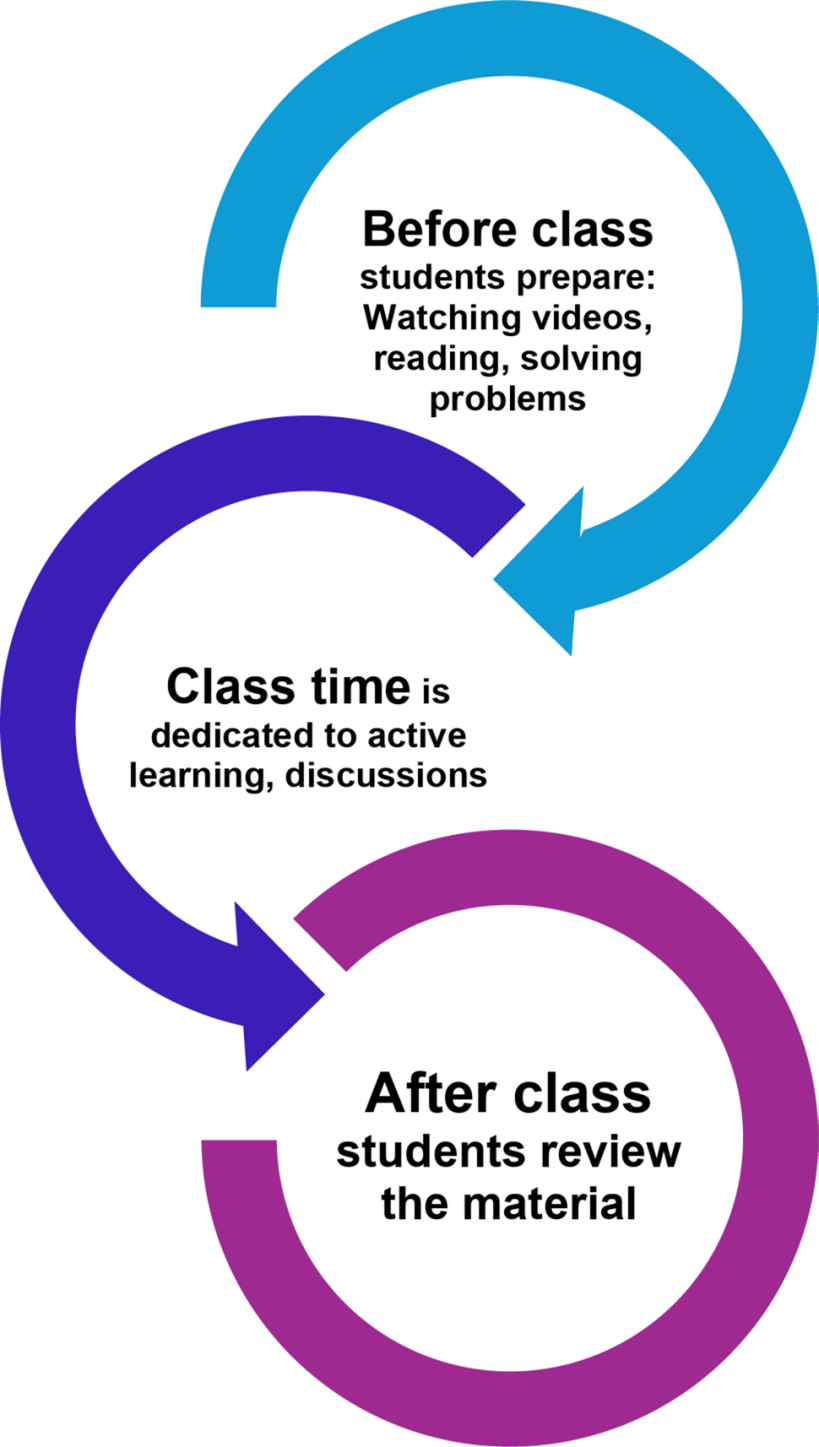
Flipped classroom model requires before, during and after class involvement

The flipped classroom model represents a transformative approach to teaching and learning, in which traditional lecture content is delivered outside of class time, such as videos, book chapters, papers, or online modules [4]. This would allow educators to use class sessions for more interactive, student-centered learning experiences. Pre-class assignments play a critical role in the flipped classroom structure. Learners are tasked with materials such as watching lectures, reading selected materials, or completing online quizzes that introduce fundamental concepts prior to lecture (Fig. 1). Students would arrive with a baseline understanding, ready to engage in more advanced application of knowledge. During the lecture, the focus is on active learning through problem-solving exercises, case discussions, collaborative group projects, and even hands-on activities. This would encourage students to apply and deepen their understanding of contents before the class in a dynamic environment.

In the flipped classroom system, learners are in the center and information flows both ways. Contrary, in traditional teaching information only flows one way from educator to learner. This model allows a learning environment where learners are empowered to take ownership of their learning journey. Deeper comprehension is possible through interaction, discussion, and applied practice.

The flipped classroom model offers numerous benefits. It enhances learners’ engagement and allows for self-paced learning. Additionally, it supports critical thinking and problem-solving skills, encourages peer collaboration, and maximizes class time for higher-order learning tasks in Blooms taxonomy including creating [5].

Although there are many advantages, flipped classroom model also has several challenges. Extensive planning and preparation of easy-to-follow, high quality resources before the class are required. Sometimes it is difficult to support learners to adapt self-directed learning which requires self-motivation [4]. Furthermore, the potential for uneven participation presents a risk of demotivating engaged learners. Basically, the flipped classroom model delivers an interactive and personalized learning experience that aligns with all levels of Bloom’s Taxonomy, inherently prioritizing active learning.

### 2—Case-based learning

Case-based learning is a widely employed method in radiology education, where educators, often attending physicians in the reading room or lecture setting, present and discuss real-life clinical cases with residents or fellows. The goal is to prepare learner for real life problems. Learners individually or in a group try to solve the problem with the guidance of educator who can answer their questions or ask more questions [6]. While case-based learning offers significant benefits, it requires a foundational understanding of the subject matter. Additionally, structured time management during sessions is essential for achieving learning goals. The strength of this approach lies in its ability to engage learners across various levels of Bloom’s Taxonomy, prompting them to recall, comprehend, and critically apply knowledge to unfamiliar scenarios. This method aligns well with the new Diagnostic Radiology Oral Certifying Exam, as it necessitates residents to articulate their detailed reasoning and formulate a diagnosis or recommendation.

### 3—Peer-teaching

Traditional radiology education typically relies on attending teaching residents or fellows during lectures or in the reading room, as well as on self-study through textbooks, online resources, or recorded lectures. However, a non-traditional approach—peer teaching—has become increasingly common in radiology training. In this method, residents at the same level teach their co-residents on specific topics or cases, either in a lecture format or through case discussions [7].

Peer teaching offers several advantages. Resident instructors are familiar with the background knowledge of their peers and can communicate using language and examples that resonate more effectively, fostering better engagement. Learners benefit from explanations that are often more relatable and delivered in a comfortable, supportive environment. Studies have shown that peer-led sessions can match or even exceed traditional teaching in terms of learner satisfaction and knowledge retention [8–10]. Additionally, residents who take on teaching roles often develop a deeper understanding of the subject matter, addressing higher levels of Bloom’s taxonomy and promoting long-term retention.

Despite its benefits, peer teaching has many challenges, primarily due to the limited teaching experience of resident educators, which can lead to misinformation or suboptimal delivery. However, these can be minimized with faculty oversight, clear feedback mechanisms, and the use of standardized educational materials. Active involvement of experienced educator in planning and supervising peer-teaching sessions ensures content accuracy and reinforces core learning objectives.

### 4—Audience response systems

ARS are increasingly utilized in radiology education to promote interactive, learner-centered teaching. These systems allow participants to respond to questions in real time using wireless keypads or web-based platforms, encouraging active engagement and providing opportunities for all learners, including those less inclined to speak publicly, to contribute. ARS enhance attentiveness, reinforce knowledge retention, and support a psychologically safe learning environment through anonymous participation. They also allow educators to assess understanding immediately, clarify misconceptions, and change their method accordingly. The ability to track responses and collect data further supports educational research and continuous improvement. In radiology, ARS can be particularly effective during case-based sessions, fostering diagnostic reasoning and promoting discussion around imaging findings. With components such as a base station, input devices, response software, and online access, ARS offer flexible, scalable solutions that enrich both in-person and remote learning experiences.

Poll Everywhere and Nearpod are popular ARS that enable educators to effectively engage their audience [11] (https://nearpod.com, https://www.polleverywhere.com). Nearpod is feature-rich with interactive question types, gamified quizzes, strong educational integrations, and real-time analytics—but requires user accounts and a subscription for full access. Poll Everywhere is easier to access (no accounts needed), integrates well with presentation tools, and supports versatile questions, though advanced analytics and large-group use require a paid plan (Table 2).

**Table 2:**
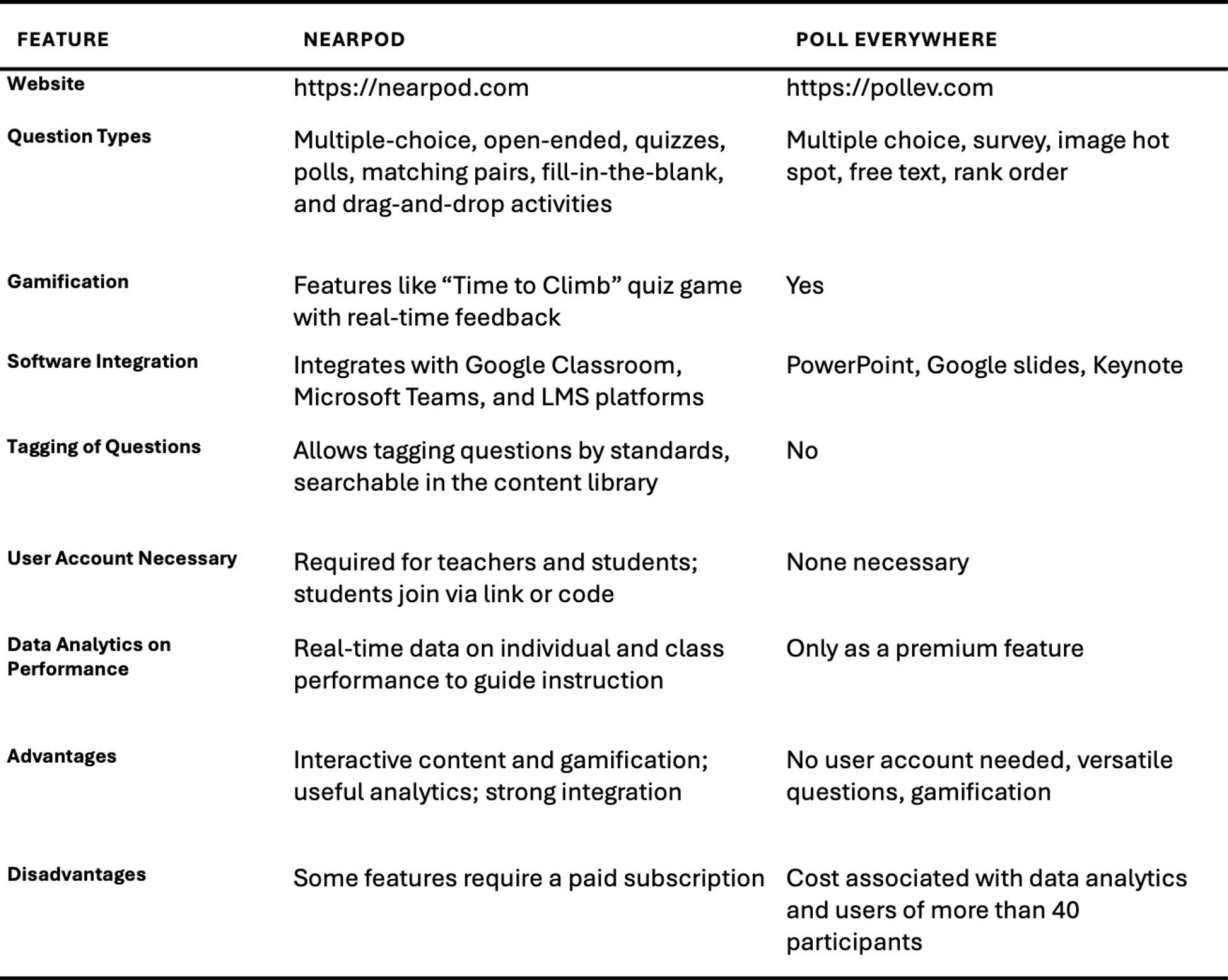
Comparison of widely used ARS in radiology education: nearpod and poll everywhere

### 5—Gamification

Gamification refers to the use of game design elements in non-game contexts with the goal of enhancing participation and user engagement [12]. In medical education, gamification is emerging as a promising tool to improve learning, engagement, and cooperation while increasing learner satisfaction [13]. Incorporating game elements such as point scoring, leaderboards, and timed challenges allows educators to transform passive learning experiences into interactive and rewarding learning environments. In radiology education, various gamified platforms are used to simulate diagnostic decision making and promote active learning [14]. Examples of gamified platforms used in radiology include but are not limited to Kaizen Education, Second Life, SonoGames, RADGames, RapRad, Kahoot, and ARS [14]. These platforms utilize various strategies from 3D virtual world designs for anatomy education to audience response systems integrated within resident lectures to improve both learner engagement and academic performance [14]. Studies have shown that these strategies are particularly effective among millennial and generation Z learners who are accustomed to digital interactivity [14–16]. However, the introduction of gamified elements must be aligned with educational objectives to promote effective learning. Gamification in education can sometimes delay learning when game elements such as chats, forums, or customization features become distractions rather than tools that enhance educational outcomes. Features like leaderboards may also demotivate lower-performing students, potentially exacerbating disparities in engagement. Moreover, many studies evaluating gamification lack strong results, most are descriptive, justification, or clarification studies, often without appropriate control groups. This makes it difficult to determine whether observed improvements in academic performance are directly attributable to gamification itself or to other confounding factors [17,18]. Future implementation of gamification in radiology should maintain the level of detail and rigor required for radiology training while incorporating the entertainment and motivational benefits of game elements.

### 6—Simulation-based learning

Simulation-based education has transformed numerous fields by providing a structured, risk-free environment where users can develop and refine their methods, enhance procedural skills, and strengthen decision-making abilities. Many industries have been impacted, such as aviation, where flight simulations are essential for pilot training. In medicine high-fidelity simulators, digital platforms, and immersive virtual reality environments provide opportunities for hands-on practice without compromising patient safety. For example, in endovascular procedures, virtual reality simulators such as the ANGIO Mentor and Vascular Intervention System Trainer (VIST) offer promising platforms for teaching simulation-based endovascular interventions to trainees [19,20].

The primary goal of simulation in radiology education is to replicate real-world clinical scenarios and procedures, allowing trainees to refine their skills in a controlled setting [21]. There are various types of simulations employed in radiology training. Diagnostic imaging simulations help residents interpret radiological studies, improving their pattern recognition and diagnostic reasoning. Procedural simulations replicate interventional techniques such as biopsies, injections, and fluoroscopy-guided procedures, enhancing technical proficiency. Additionally, call-taking simulations, often web-based, allow trainees to experience on-call scenarios, helping them build confidence in managing urgent radiological findings.

**Fig. 2:**
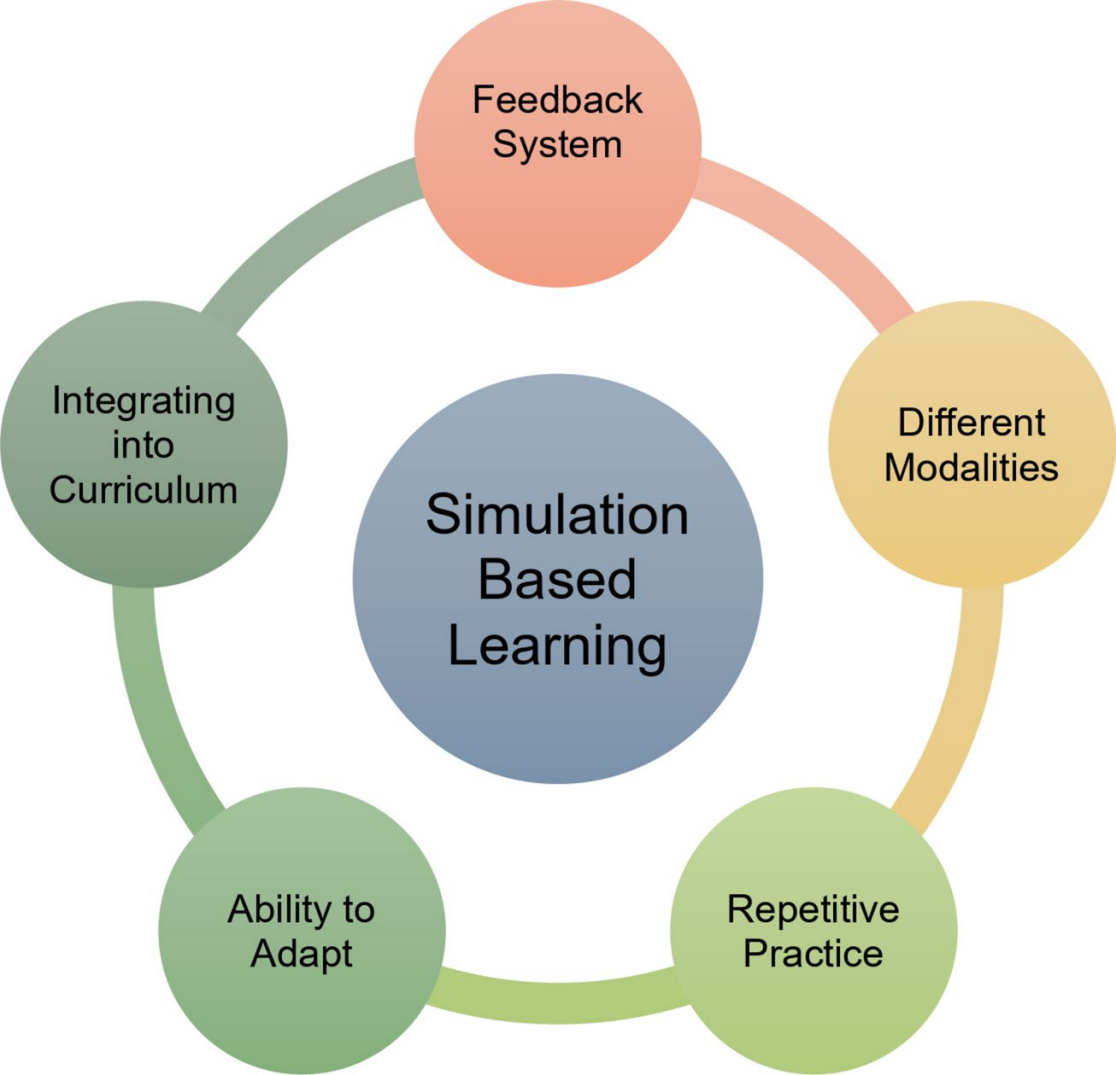
Adapting simulation-based learning (SBL) into the curriculum requires multiple steps, including establishing a feedback system, incorporating diverse modalities, allowing for repetitive practice, and possessing the ability to adapt

The integration of simulation into radiology education serves multiple objectives and can be implemented at various stages of training (Fig. 2). During residency, structured simulation-based curricula refine both diagnostic and procedural expertise. A common example of simulation in residency training is first-year residents reading out cases with an attending radiologist. During these sessions, residents present and discuss imaging findings before finalizing reports, simulating real-world decision-making while receiving direct mentorship. Another example involves creating a simulation of an image-guided biopsy for residents to practice before performing the actual procedure on patients.

Despite its many advantages, simulation-based education in radiology presents certain challenges. Developing and maintaining high-quality simulation programs requires significant financial and infrastructural investment. Faculty training is essential to ensure effective simulation-based instruction, yet many institutions struggle with limited resources. Additionally, integrating simulation into already demanding residency schedules can be complex, requiring careful planning to balance clinical responsibilities with educational activities. Another critical consideration is ensuring the validity and reliability of simulation scenarios to maintain educational effectiveness [21].

### 7—AI and teaching

Artificial intelligence (AI) is increasingly being utilized in radiology education to enhance educational outcomes within the field [22]. Prior to the introduction of AI, radiology education centered on rigorous anatomy training, hands-on practice with various imaging modalities, and clinical problem-solving with complex cases [23]. However, these methods are limited by availability of educational cases and variability between teaching radiologists [24]. The emergence of AI-powered platforms can now simulate real-life diagnostic challenges using extensive annotated datasets, which allows learners to interact with complex imaging cases in a personalized manner [25]. These systems are designed to provide immediate and personalized feedback which enables to learner to improve without direct faculty supervision [25]. There are numerous examples of AI-powered platforms developed specifically for medical education, as many institutions are developing their own models and software to integrate AI into teaching workflows. Platforms such as Stanford University’s Artificial Intelligence in Medical Imaging (AIMI) Center provides open-access annotated imaging datasets and research tools that facilitate hands-on AI training for medical students and radiology residents. Other tools, like the Adaptive Radiology Interpretation and Education System (ARIES), is an open-source software that integrates clinical and imaging data to teach probabilistic reasoning in forming differential diagnoses [26]. These developments expand access to high-quality educational materials and allow for standardization in learning experiences across different training environments. Despite its promise, integrating AI into radiology education requires careful oversight, as early reliance may hinder trainees from developing essential interpretive skills. Inexperience can increase susceptibility to automation bias, emphasizing the need for structured integration of AI tools at appropriate stages of training [27]. AI and large language models (LLMs) have raised concerns that students may become overly dependent on these tools, potentially leading to a loss of independent critical thinking and a decline in active learning skills [28]. As AI technology continues to advance, programs should teach trainees to critically assess AI-generated results and understand their limitations while encouraging involvement in quality improvement initiatives that promise the refinement of AI tools.

### 8—Role for VR

Virtual reality (VR) is emerging as a novel educational tool in radiology that offers immersive three-dimensional environments for interactive learning [29]. VR platforms offer the ability to simulate an environment and interact with spatial relationships that are challenging to convey through traditional two-dimensional models [29]. VR has been utilized in medicine since the 1990s to aid in procedural training and revitalize educational experiences. In education, VR has been shown to be effective in helping students master radiographic technique, cooperate with virtual patients, and understand complex anatomy through immersive, three-dimensional exploration [30]. In interventional radiology, VR serves as a valuable tool for procedural training. It provides a risk-free environment where trainees can practice techniques such as central venous catheter placement [31] and videoscopic phantom-based angiographic simulation—procedures that require careful spatial orientation and real-time decision making without the consequences of patient harm [32]. Moreover, VR has been utilized to create virtual radiology workstations, allowing trainees exposure to imaging studies in a simulated Picture Archiving and Communications Systems (PACS) environment [33]. This is particularly beneficial as it promotes the development of diagnostic skills in an interactive and controlled setting [33]. Similarly to gamification elements, the immerse nature of VR encourages learner engagement and may lead to improved knowledge retention compared to conventional teaching methods [34]. While the future of VR in radiology education seems promising, the technology still faces several challenges. These include high costs, limited access to VR hardware across institutions, and the need for faculty training and integration within the training curriculum [35]. In the future, further validation studies are needed to determine the long-term education efficacy of VR technology and maintain consistent learning outcomes across different learning environments.

## Discussion

Each teaching strategy brings its own unique strengths and limitations, highlighting the importance of tailored integration into radiology education based on institutional resources, educator availability, and learner needs. An important point is the necessity of incorporating continuous feedback mechanisms. These mechanisms should not be confined to simulation-based learning alone, but embedded across all teaching strategies to both motivate learners and enable educators to adaptively refine instructional design. Continuous assessment is essential to evaluate not just learner progress, but also the efficacy of the educational programs themselves. Also another limitation is teaching staff availability, especially in radiology with growing workload where faculty often has limited time, plays a crucial role in determining the optimal strategy. For instance, the flipped classroom approach can be highly efficient when faculty time is limited, allowing learners to absorb foundational knowledge independently and reserving sessions for higher level discussions including clinical reasoning. When attending radiologist is not available, peer-teaching offers a pragmatic alternative, especially when coupled with standardized materials and oversight to ensure educational integrity.

The success of any teaching strategy depends on underlying curriculum. Structured frameworks ensure comprehensive coverage of clinically relevant scenarios. Without such curricular scaffolding, even the most advanced pedagogical tools risk being underutilized or misaligned with clinical practice needs. Ultimately, the integration of diverse teaching strategies should be guided by their alignment with educational objectives, logistical feasibility, and the dynamic feedback loops between learners and educators.

## Conclusion

The evolution of radiology education from traditional didactic methods to technologically driven, interactive learning approaches represents a significant advancement in medical training. The integration of AI, VR/AR, simulation-based learning, and active learning models would enhance learning ability, critical thinking, and hands-on proficiency. However, successful implementation requires addressing timing, financial, logistical, and faculty training challenges. As medical education continues to evolve, embracing these innovative strategies will ensure that radiologists are well-equipped to meet the demands of modern healthcare.

## Data Availability

No datasets were generated or analysed during the current study.
